# Supplementary material for: Amyloid aggregates accumulate in melanoma metastasis modulating YAP activity
Source: EMBO Rep. 2020 Aug 4;21(9):e50446. doi: 10.15252/embr.202050446 (PMC7507035; doi:10.15252/embr.202050446)
Supplement: Supplementary file 1 — Expanded View Figures PDF [file EMBR-21-e50446-s001.pdf]

Expanded View Figures

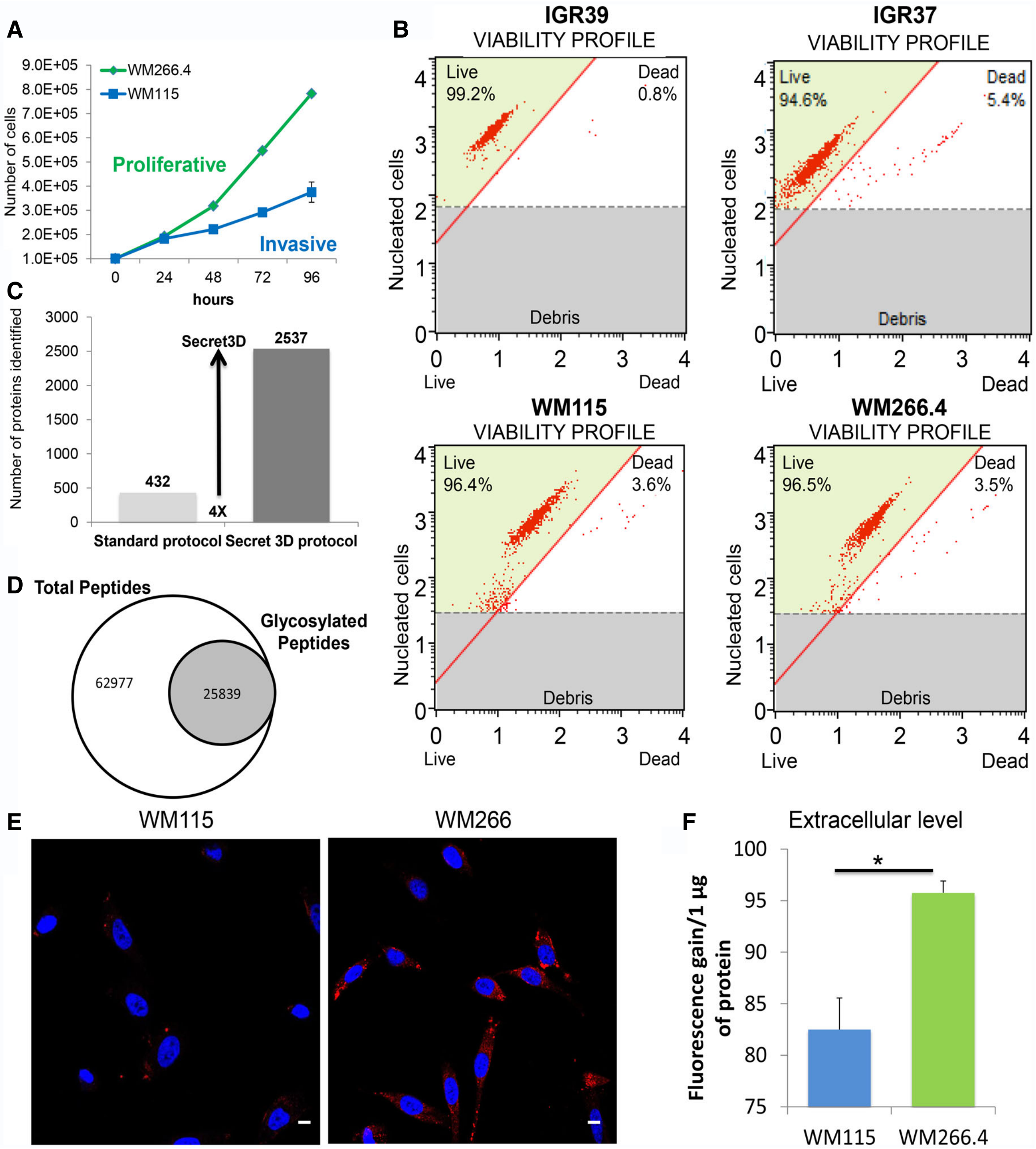

Figure EV1. (Related to Figs 1 and 2) Analysis of a model system for primitive and metastatic melanoma, Secret3D and Proteostat analysis.

- A Growth curve of primitive WM115 and metastatic WM266.4 cell line (N = 3 biological replicates) measured as number of cells/time.
- B Viability profile of IGR and WM cell lines after 24-h starvation, detected by measuring cell confluence (%) and number of dead and alive cells using Muse™ Cell Analyzer.
- C Number of proteins identified in the secretome by Secret3D compared to a standard protocol.
- D Number of total and glycosylated peptides identified by Secret3D.
- E Confocal immunofluorescence images of Proteostat (1:2,000, red spots) and DAPI staining (blue) for WM115 and WM266.4 cells. Scale bar is 10 µm.
- F Quantitation of aggregates in the extracellular space in WMs. Fluorescence gain of proteins present in the supernatant treated with Proteostat reagent. (*T*-test, *P < 0.05) N = 4 biological replicates. Data are mean ± SD.

© 2020 The Authors

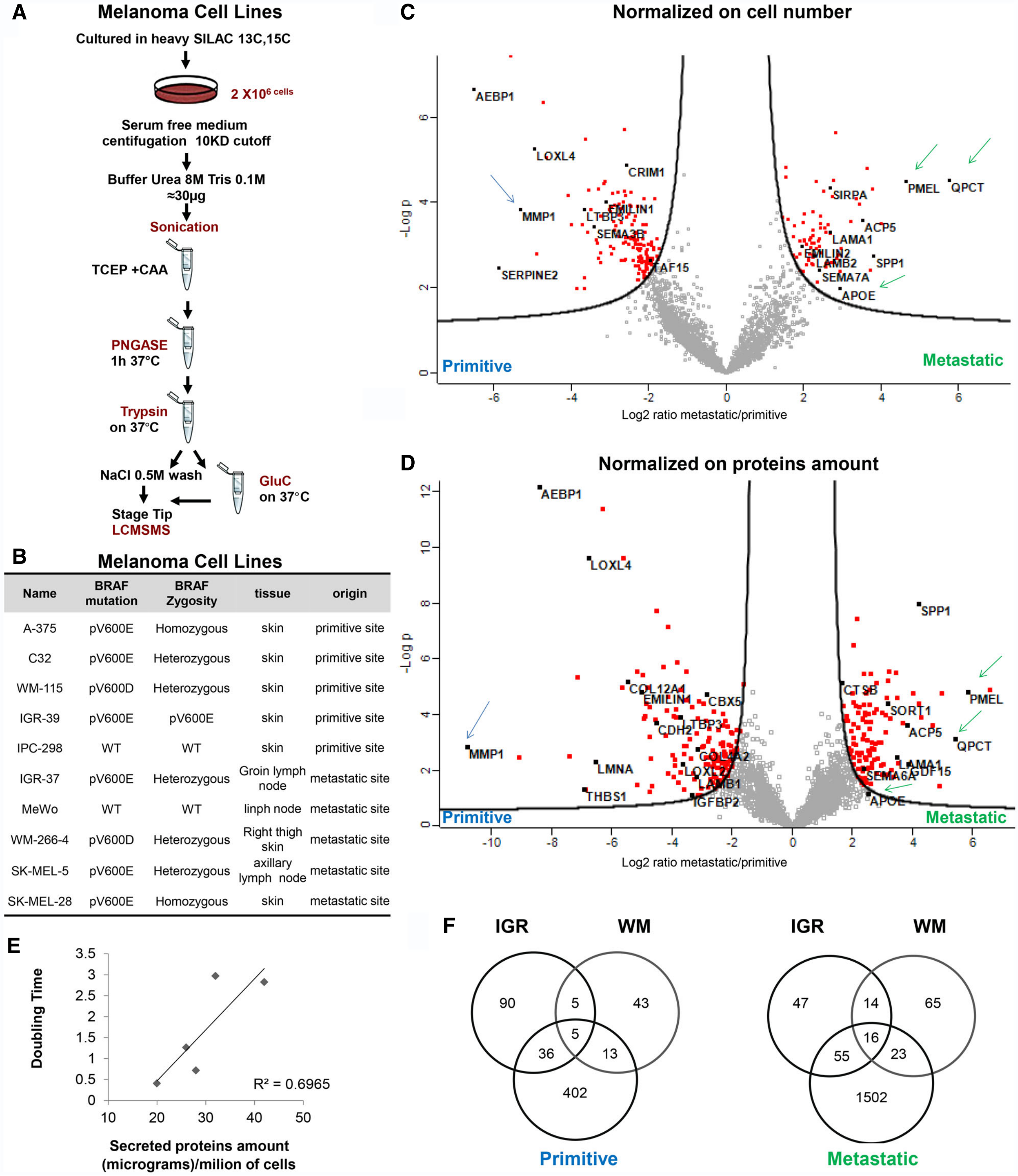

Figure EV2. (Related to Fig 2) Proteomic analysis of the secretome from primitive and metastatic melanoma cells.

A MS workflow of Secret3D applied on melanoma cell lines described in (B).

B Cell lines used in this study. Their origin and BRAF mutation status are reported.

C, D Secretome analysis of A375, C32, IPC298, SKMEL5, SKMEL28, MEWO melanoma cell lines described in (B). Volcano plot of the quantified proteins with both normalization strategies discussed in the main text. Green arrows indicate proteins overexpressed in metastatic cells, and blue arrows indicate proteins overexpressed in primary cells.

E Scatter plot of secreted proteins versus doubling time of the cell lines analyzed.

F Venn diagram of the significant proteins shared by all melanoma cell lines.

© 2020 The Authors

Figure EV3. (Related to Figs 3 and 4) Gene expression and protein aggregates level in primitive vs. metastatic melanoma cell lines. Mosaic acquisitions of melanoma metastases. Expression profiling by cancer type and overall survival analysis for BACE2 in melanoma patients. Cell viability in IGRs upon BACE inhibition.

A Gene expression data from the Cancer Cell Line Encyclopedia (CCLE). Gene expression level for the selected genes was searched in the same melanoma cell lines used for proteomic analysis. Primitive melanoma cell lines were WM115, IGR39, A375, C32, and IPC298, while metastatic melanoma cell lines were WM266.4, IGR37, SKMEL5, SKMEL28, and MEWO. *T*-test, *$P < 0.05$, **$P < 0.001$. Data are expressed as mean ± SD.

B Analysis of protein aggregates present in the secretome of 10 different melanoma cell lines. Fluorescence gain of proteins present in the supernatant treated with Proteostat reagent is reported. *T*-test analysis, ***$P < 0.001$. Data are mean ± SD.

C Mosaic acquisition of melanoma metastases in human brain stained with hematoxylin–eosin or with Proteostat. The arrows point at melanin signals. Scale bar is 300 μm.

D KI-67 staining (brown) in healthy tissue, primitive melanoma and metastatic melanoma as indicated. Scale bar is 50 μm.

E BACE2 expression profiling by cancer type in TCGA normal and GTEx dataset by using GEPIA software (http://gepia.cancer-pku.cn/). The gene expression profile across all tumor samples (red dots) and paired normal tissues (green dots). Each dot represents expression of samples.

F Overall survival analyses performed using the GEPIA online platform for melanoma dataset. The solid line represents the survival curve, and the dotted line represents the 95% confidence interval. Log-rank $P < 0.05$ was considered to indicate a statistically significant difference. Patients with expression above the median are indicated by red lines, and patients with expression below the median are indicated by blue lines. BACE2 expression level (Transcript Per Million, TPM) is negatively associated with the overall survival of melanoma patients.

G Gene expression level (Transcript Per Million, TPM) in cancer/normal tissue for the selected genes extracted from TCGA normal and GTEx data in melanoma dataset contained in GEPIA software. *T*-test: *$P < 0.05$.

H Cell viability of IGR cell lines treated with NB-360 at different concentrations and time of incubation as reported. $N = 3$, *T*-test analysis. **$P < 0.01$; ***$P < 0.001$. The histogram represents total cell number. The horizontal line represents % of alive cells.

I Pigmentation of IGR37 upon incubation with NB-360. Red box shows depigmentation after NB-360 administration.

 © 2020 The Authors

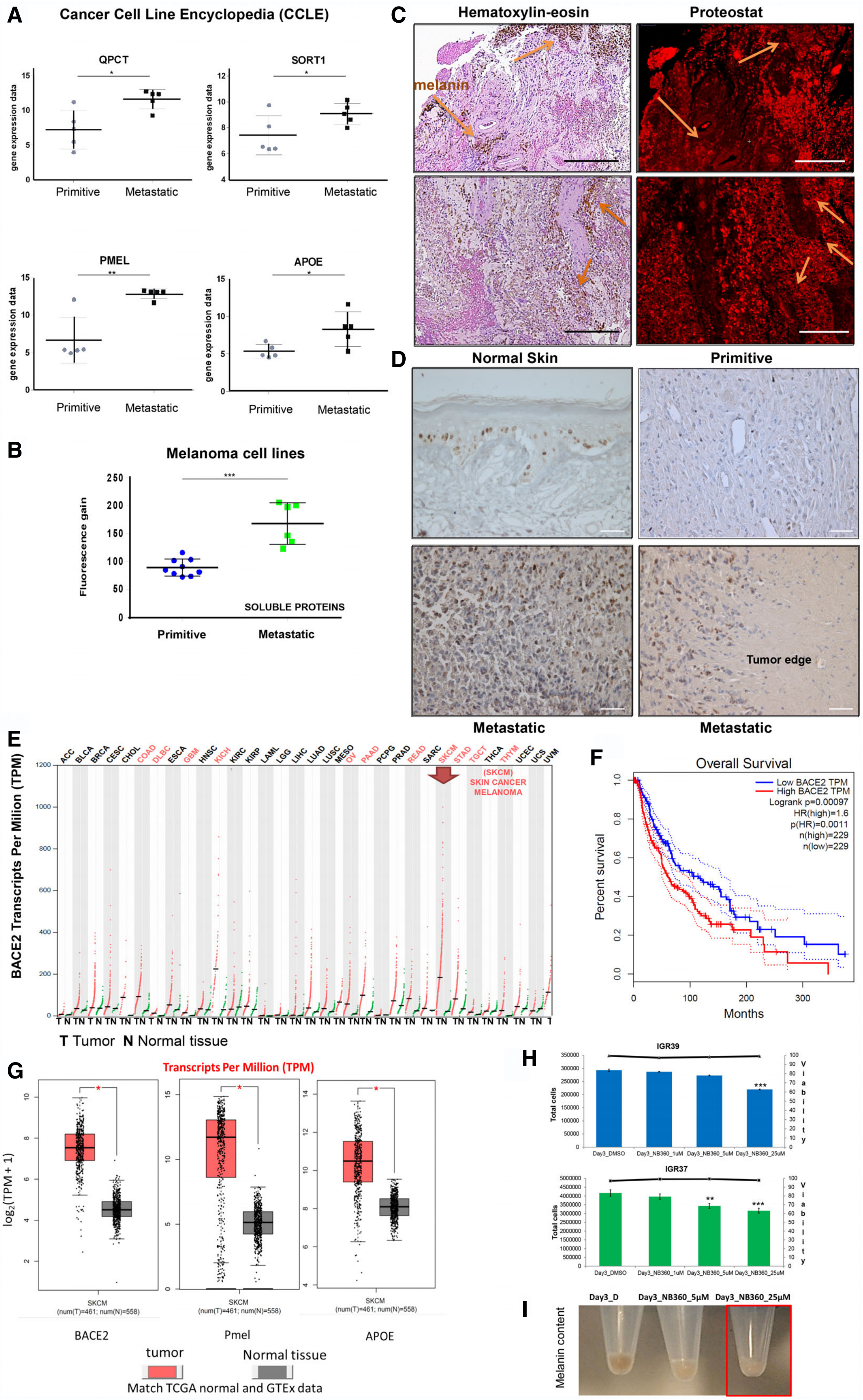
A
Cancer Cell Line Encyclopedia (CCLE)
QPCT
SORT1
PMEL
APOE
gene expression data
Primitive
Metastatic
B
Melanoma cell lines
Fluorescence gain
SOLUBLE PROTEINS
C
Hematoxylin-eosin
Proteostat
melanin
D
Normal Skin
Primitive
Tumor edge
Metastatic
E
BACE2 Transcripts Per Milion (TPM)
(SKCM)
SKIN CANCER
MELANOMA
T Tumor N Normal tissue
F
Overall Survival
Low BACE2 TPM
High BACE2 TPM
Logrank p=0.00097
HR(high)=1.6
p(HR)=0.0011
n(high)=229
n(low)=229
Percent survival
Months
G
Transcripts Per Million (TPM)
BACE2
Pmel
APOE
tumor
Normal tissue
Match TCGA normal and GTEx data
H
IGR39
IGR37
Total cells
Viability
I
Day3_D
Day3_NB360_5μM
Day3_NB360_25μM
Melanin content

Figure EV3.

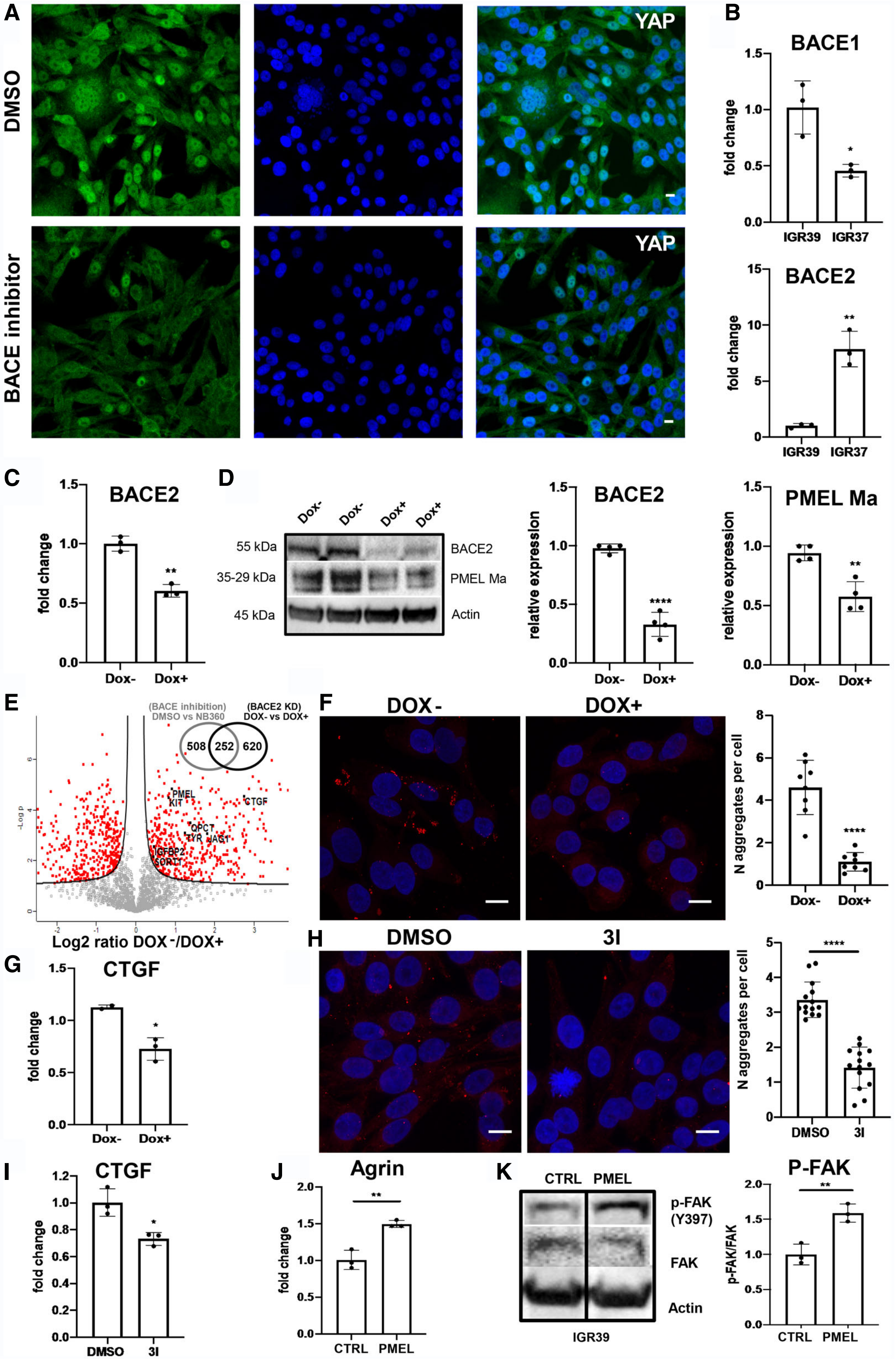

Figure EV4.

© 2020 The Authors

Figure EV4. (Related to Fig 5) Effect of BACE1/2 inhibition and BACE2 KD in IGR37 metastatic melanoma cells and of PMEL administration in IGR39 primitive cells.

A Confocal fluorescence images of anti-YAP antibody (green) and DAPI staining (blue) in IGR37 upon treatment with DMSO or BACE1/2 inhibitor. Scale bar is 10 μm.

B BACE1 and BACE2 mRNA levels measured by real-time PCR in IGR37 and IGR39. $N = 3$ biological replicates. *T*-test, $*P < 0.05$, $**P < 0.001$. Data are mean ± SD.

C BACE2 mRNA levels measured by real-time PCR in iBACE2 KD IGR37 treated or not with doxycycline. $N = 4$ biological replicates. *T*-test analysis, $**P < 0.001$. Data are mean ± SD.

D Western blot and densitometric analysis of BACE2 and PMEL Mα relative expression in iBACE2 KD IGR37 treated or not with doxycycline. Actin was used as loading control. $N = 4$ biological replicates. *T*-test, $**P < 0.01$, $****P < 0.0001$. Data are mean ± SD.

E Volcano plot of the proteins secreted by iBACE2 KD IGR37 cells treated or not with doxycycline (Secret3D workflow). $N = 4$ biological replicates. Proteins downregulated upon BACE2 silencing are reported in the right part of the plot. Venn diagram of statistically significant proteins common between NB-360 treatment and BACE KD in IGR37 (right upper corner).

F Confocal fluorescence images of Proteostat signal (1:2,000, red) and DAPI staining (blue), scale bar is 10 μm, and quantitation of protein aggregates in iBACE2 KD IGR37 cell lines treated or not with doxycycline by immunofluorescence analysis using Fiji software. $N = 8$ biological replicates. *T*-test, $****P < 0.0001$. Data are mean ± SD.

G mRNA levels of CTGF measured by real-time PCR in iBACE2 KD IGR37 treated or not with doxycycline. $N = 3$ biological replicates. *T*-test, $*P < 0.05$. Data are mean ± SD.

H Confocal fluorescence images of Proteostat signal (1:2,000, red) and DAPI staining (blue), scale bar is 10 μm, and quantitation of protein aggregates in IGR cell lines treated or not with 3I inhibitor by immunofluorescence analysis using Fiji software. $N = 14$ biological replicates. *T*-test, $****P < 0.0001$. Data are mean ± SD.

I mRNA levels of CTGF measured by real-time PCR in IGR37 treated or not with 3I inhibitor. $N = 3$ biological replicates. *T*-test analysis, $*P < 0.05$. Data are mean ± SD.

J mRNA levels of Agrin measured by real-time PCR in IGR39 treated with recombinant PMEL. $N = 3$ biological replicates. *T*-test, $**P < 0.01$. Data are mean ± SD.

K Western blot and densitometric analysis of pFAK(Y397) in IGR39 treated with recombinant PMEL. Actin was used as loading control. $N = 3$ biological replicates. *T*-test, $**P < 0.01$. Data are mean ± SD.

Source data are available online for this figure.

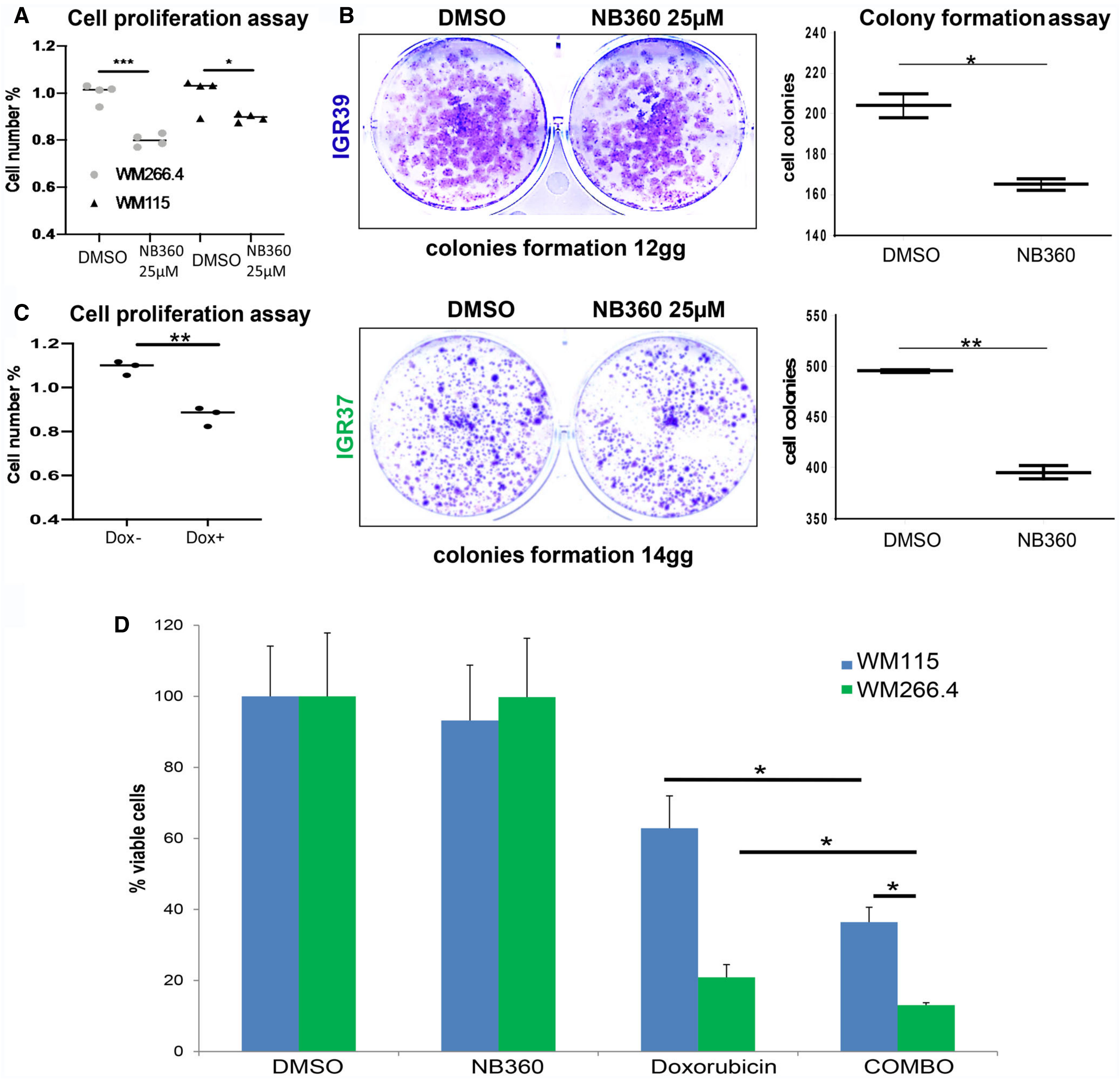

Figure EV5. (Related to Fig 6) Effect of BACE inhibition and BACE2 KD on colony formation, cell proliferation, and chemo-sensitivity.

A MTT assay for WMs treated with DMSO or NB-360 (25 μM). N = 4 biological replicates. *T*-test analysis, *$P < 0.05$, ***$P < 0.001$. Data are mean ± SD.

B Colony formation assay and relative quantitation on the right panels, for IGR39 and IGR37 cells treated with DMSO or NB-360. N = 3 biological replicates. *T*-test analysis, *$P < 0.05$, **$P < 0.01$. Data are mean ± SD.

C MTT assay of iBACE2 KD IGR37 cells treated or not with doxycycline. N = 3 biological replicates. *T*-test analysis, **$P < 0.01$. Data are mean ± SD.

D Viability of WM melanoma cell lines treated with DMSO, doxorubicin 10 μM, NB-360 25 μM, and the combination of doxorubicin and NB-360 measured as % of viable cells. N = 3 biological replicates. *T*-test analysis, *$P < 0.05$. Data are mean ± SD.

© 2020 The Authors
