# Supplementary material for: Amyloid aggregates accumulate in melanoma metastasis modulating YAP activity
Source: EMBO Rep. 2020 Aug 4;21(9):e50446. doi: 10.15252/embr.202050446 (PMC7507035; doi:10.15252/embr.202050446)
Supplement: Supplementary file 13 — Movie EV1 [file EMBR-21-e50446-s013.zip › Movie EV1 legend.docx]

**Movie EV1. Representative Time lapse imaging of living IGR39 cells**. The images were collected every 5 min for a total recording time of 72 h.
