# Supplementary figures and images for: Amyloid aggregates accumulate in melanoma metastasis modulating YAP activity
Source: EMBO Rep. 2020 Aug 4;21(9):e50446. doi: 10.15252/embr.202050446 (PMC7507035; doi:10.15252/embr.202050446)

Source data Figure EV 4 D

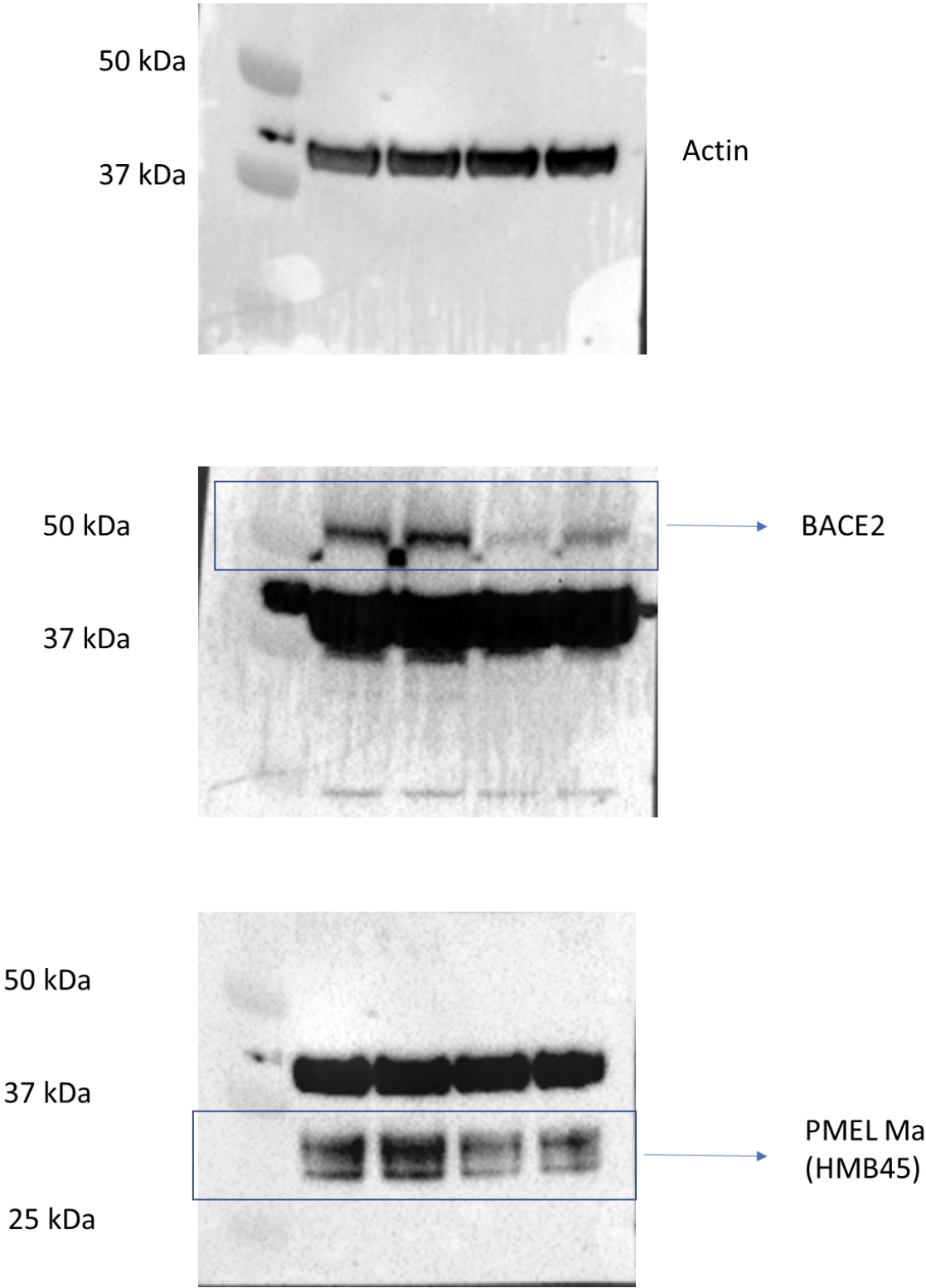

Source Data for Figure EV 4M

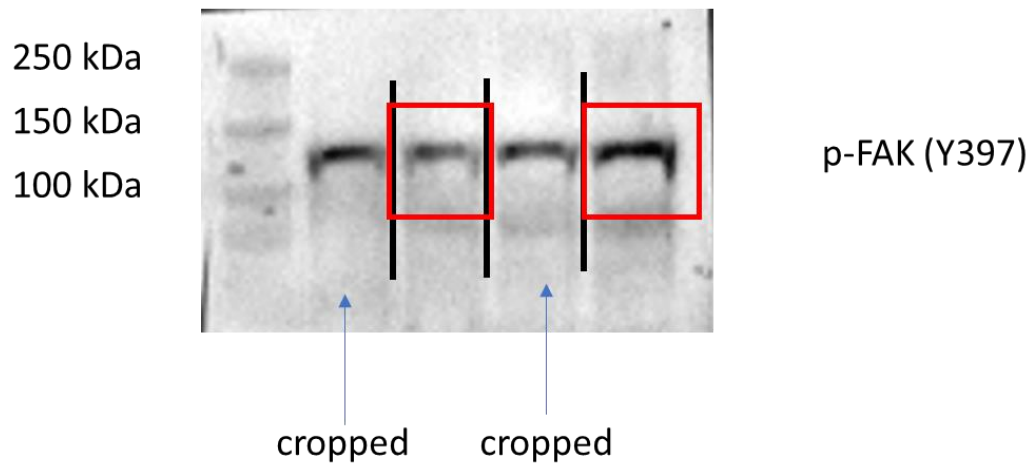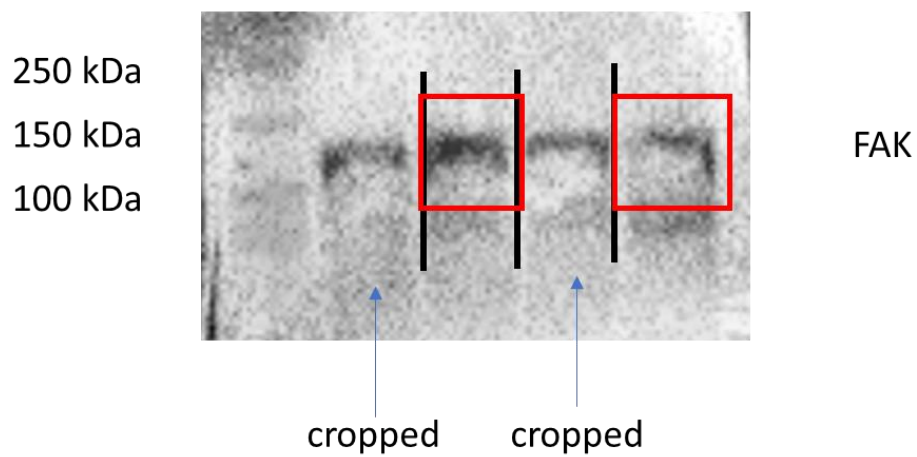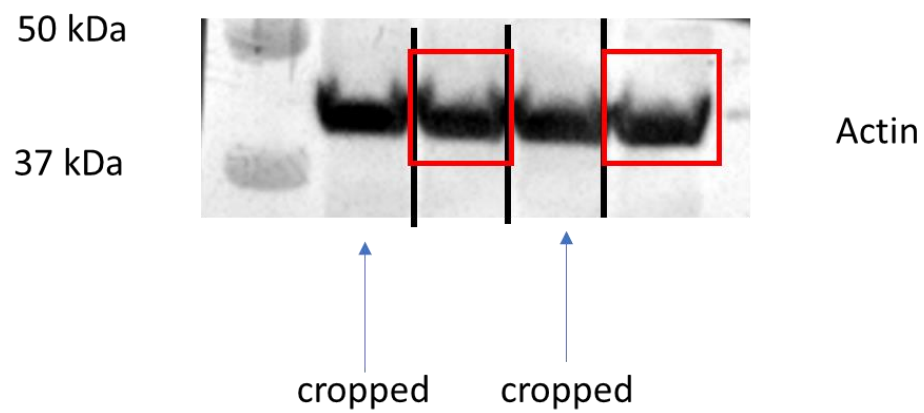

Supplement: Supplementary file 15 — Source Data for Expanded View [file EMBR-21-e50446-s016.pdf]

Source Data Figure 3 panel D

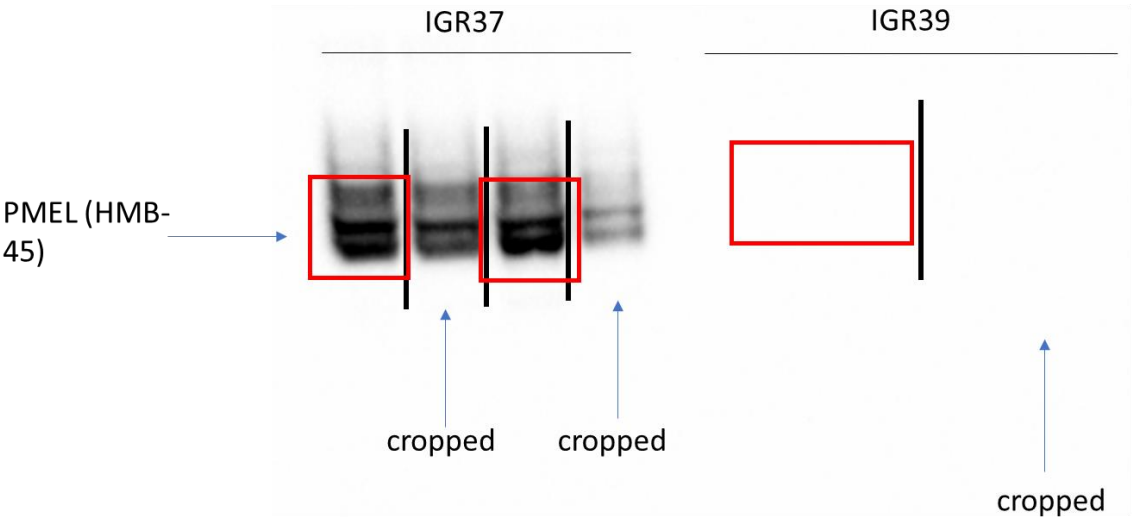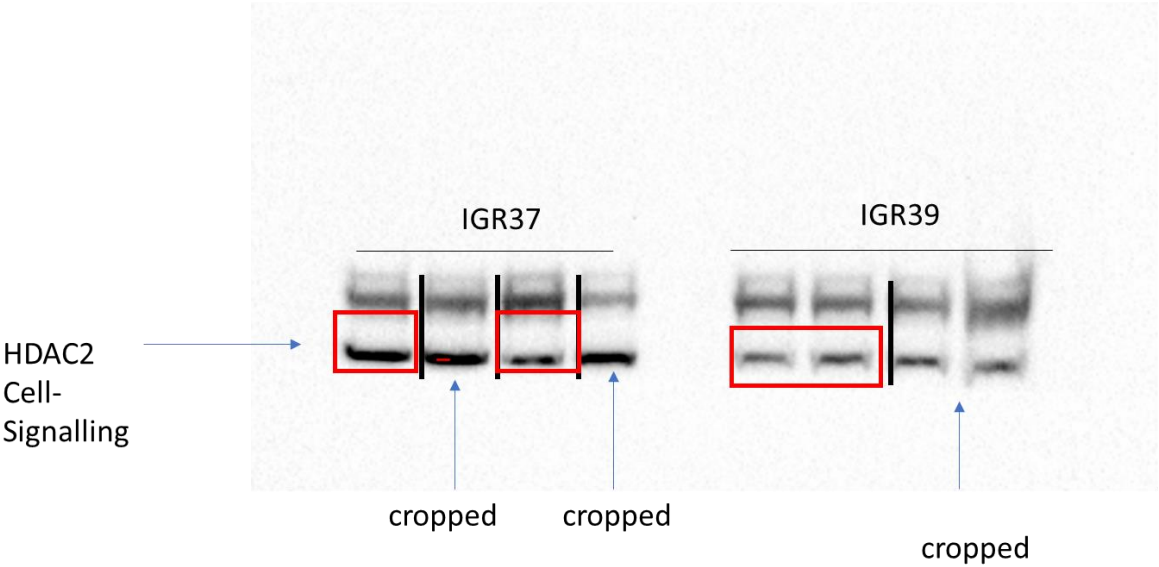

Supplement: Supplementary file 17 — Source Data for Figure 3 [file EMBR-21-e50446-s015.pdf]
